# Condensed tannins act as anthelmintics by increasing the rigidity of the nematode cuticle

**DOI:** 10.1038/s41598-022-23566-2

**Published:** 2022-11-07

**Authors:** Luise Greiffer, Eva Liebau, Fabian C. Herrmann, Verena Spiegler

**Affiliations:** 1 Institute for Pharmaceutical Biology and Phytochemistry, University of Münster, Münster, Germany; 2 Institute of Integrative Cell Biology and Physiology, University of Münster, Münster, Germany

**Keywords:** Parasitic infection, Atomic force microscopy, Natural products

## Abstract

Tannins and tanniferous plant extracts have been discussed as sustainable means for helminth control in the past two decades in response to a dramatic increase of resistances towards standard anthelmintics. While their bioactivities have been broadly investigated in vitro and in vivo, less is known about their mode of action in nematodes, apart from their protein binding properties. In the current study we therefore investigated the impact of a phytochemically well characterized plant extract from *Combretum mucronatum*, known to contain procyanidins as the active compounds, on the model organism *Caenorhabditis elegans*. By different microscopic techniques, the cuticle was identified as the main binding site for tannins, whereas underlying tissues did not seem to be affected. In addition to disruptions of the cuticle structure, molting defects occurred at all larval stages. Finally, an increased rigidity of the nematodes’ cuticle due to binding of tannins was confirmed by force spectroscopic measurements. This could be a key finding to explain several anthelmintic activities reported for tannins, especially impairment of molting or exsheathment as well as locomotion.

## Introduction

Infestations with intestinal helminths are still affecting more than one billion people worldwide, mainly in Sub-Sahara Africa, South East Asia and South America^1^. The current strategies to tackle these diseases are mass drug administration programs^1^. However, despite the fact that several hundred million preschool and school-aged children in endemic areas receive preventive treatment, benefits are not entirely clear^2,3^ and preventive deworming favours the emergence of resistances against standard anthelmintics, similar to what has been observed in veterinary medicine in the past decades^4^. One potential means for a sustainable parasite control in livestock is the use of tannin-rich forages or plant extracts to reduce worm burden or egg excretion in animals^4–6^. On the other hand, preparations and extracts from tanniferous plants are also used as anthelmintic remedies in humans within the traditional medicine of certain endemic countries^7^. Tannins comprise a subclass of polyphenolic secondary plant metabolites that can be structurally divided into two major groups, namely condensed tannins (syn. proanthocyanidins) and hydrolysable tannins (ellagitannins and gallotannins)^8,9^. Regarding their anthelmintic activity, a considerable number of studies have been conducted in vitro and in vivo in the past two decades, reporting a certain efficacy depending on tannin structure, parasite species and host animal^10–12^. What still remains unclear is the mode of action.

By their ability to bind proteins^13–16^ it seems likely that tannins generally do not address a specific target, but are able to tackle different structures of the nematodes. Due to its high content in proline-rich collagens^17,18^ which bind tannins with high affinity^19^, the cuticle seems to be a target structure easily accessible to macromolecules like tannins. Indeed, a series of microscopical investigations showed detrimental effects on the cuticle, sometimes extending to the underlying tissues, in various parasitic nematode species^11,20–25^ well as in the free-living model organism *Caenorhabditis elegans*^26,27^. Further common anthelmintic activities exerted by tannins apart from nematicidal effects include the inhibtion of egg hatch, larval motility and larval exsheathment^10^. The mechanism behind these effects has not been fully explained until now. One hypothesis is that alterations in the cuticle structure as mediated by tannin treatment could lead to a reduced flexibility^23^ and thus to the impaired motility of the nematodes^20,25^, since one major function of the cuticle is to aid locomotion^18,28^. Inhibition of exsheathment, in turn, has been associated with tannins disturbing the activity of enzymes on the larval sheath^29,30^ or, more generally, interfering with metabolic processes involved in molting^25^. Larval exsheathment is an activity frequently assayed in infectious L3 larvae of certain parasitic nematode species (e.g. *Haemonchus contortus* or *Trichostrongylus colubriformis*). However, the effect on molting after treatment with tannins has not been assessed in *C. elegans* until now, although *C. elegans* is a common model organism for nematodes^31,32^ which has also been proven useful in the discovery of novel anthelmintics^33,34^.

One aim of this study was therefore, to investigate the effects of a traditionally used plant extract from *Combretum mucronatum*^7^ containing procyanidins as the active anthelmintic compounds^35^ on molting in *C. elegans*. However, the main aim was to experimentally verify by atomic force microscopy (AFM) the hypothesis of tannins affecting the flexibility of the nematodes’ cuticle in order to contribute to a better understanding of the mode of anthelmintic action of condensed tannins.

## Results

### Treatment with a tannin rich ethanolic extract of *Combretum mucronatum* causes alterations in the cuticle structure in C. *elegans*

**Figure 1:**
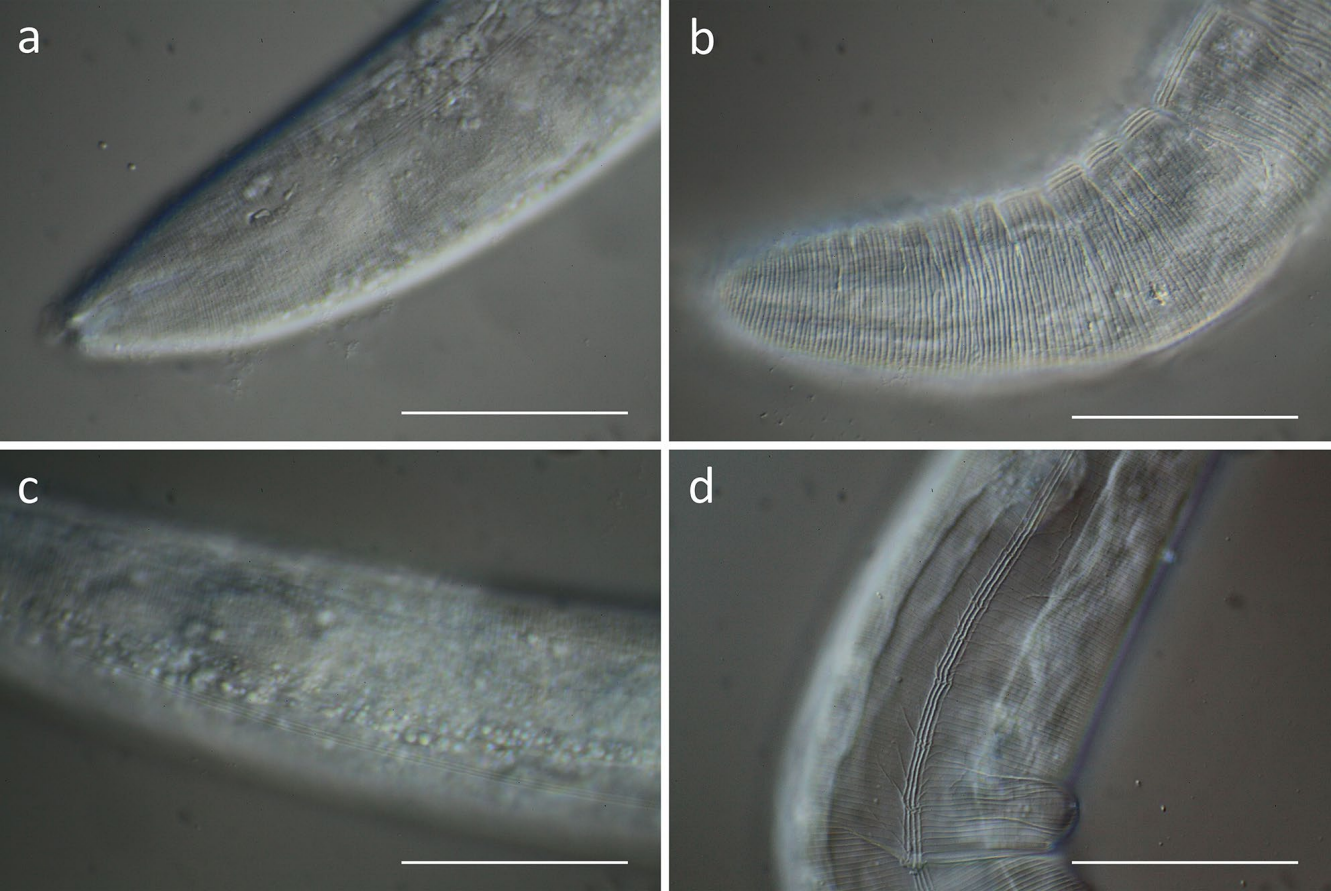
Differential interference contrast microscopy (DIC) of the cuticle surface of young adult *C. elegans* in head and mid-body region after 24 h of treatment followed by 6 h of regeneration on NGM agar. (**a**,**b**) Untreated control (M9 buffer with 1% DMSO), (**c**,**d**) worms treated with CM (2 mg/ml). Scalebar 50 µm.

The first step of the current study was to investigate structural changes to the cuticle and underlying tissues in *C. elegans* after treatment with a tanniferous hydroethanolic extract from the leaves of *C. mucronatum* (“CM”). The tannin content in CM was determined to be 21% related to the dry extract. Young adult *C. elegans* worms (N2 Bristol) were treated with CM (2 mg/mL) for 24 h. For a better visualization of potential disruptions of the cuticle and underyling tissues, worms were then allowed to regenerate and move on NGM-agar plates in order to increase mechanical forces on the cuticle which do not occur to the same extent in liquid culture^36^. DIC microscopy revealed clear alterations to the cuticle of the treated nematodes. While the control group generally showed smooth and regular structures (Fig. 1a,b), these patterns seemed shrivelled and less regular in the treated group, particularly in the head region (Fig. 1c,d). In addition to the structural effects, a reduced motility was observed after treatment.

**Figure 2:**
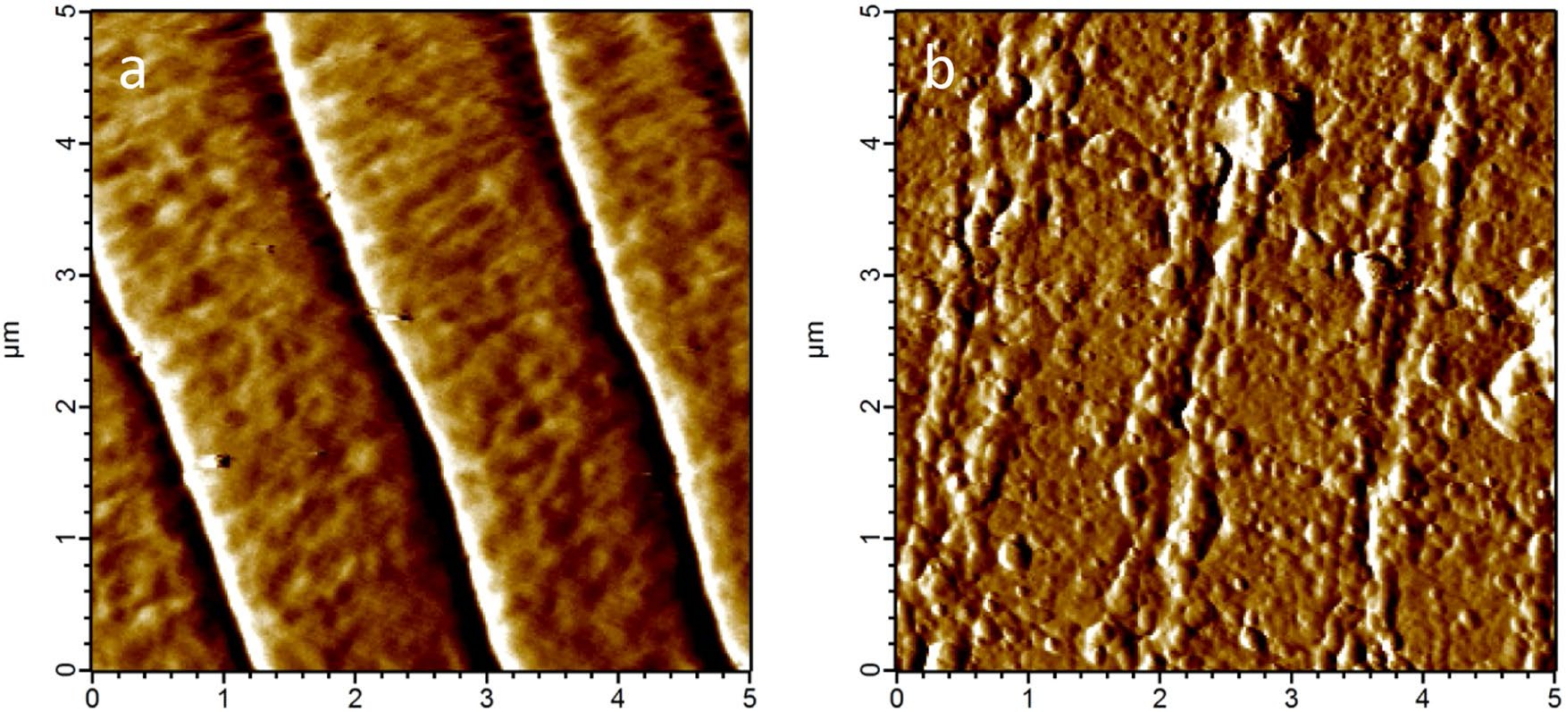
Topographic AFM images of the cuticle surface of young adult *C. elegans* after 24 h of treatment. (**a**) Untreated control, (**b**) worms treated with CM (2 mg/mL).

To investigate these cuticle alterations in more detail, atomic force microscopy (AFM) was performed to obtain topographic images of the cuticle surface of living *C. elegans* individuals. As shown in Fig. 2, the regular structure of furrows and annuli as seen in the untreated control (Fig. 2a), is clearly disturbed in the treated samples (Fig. 2b). In addition, despite repeated washing of the worms prior to image acquisition, agglomerates were found on the worms’ surface, especially in the furrows (Fig. 2b), which represent either residues of the extract, or more likely, aggregates formed by proanthocyanidins bound to cuticle proteins.

**Figure 3:**
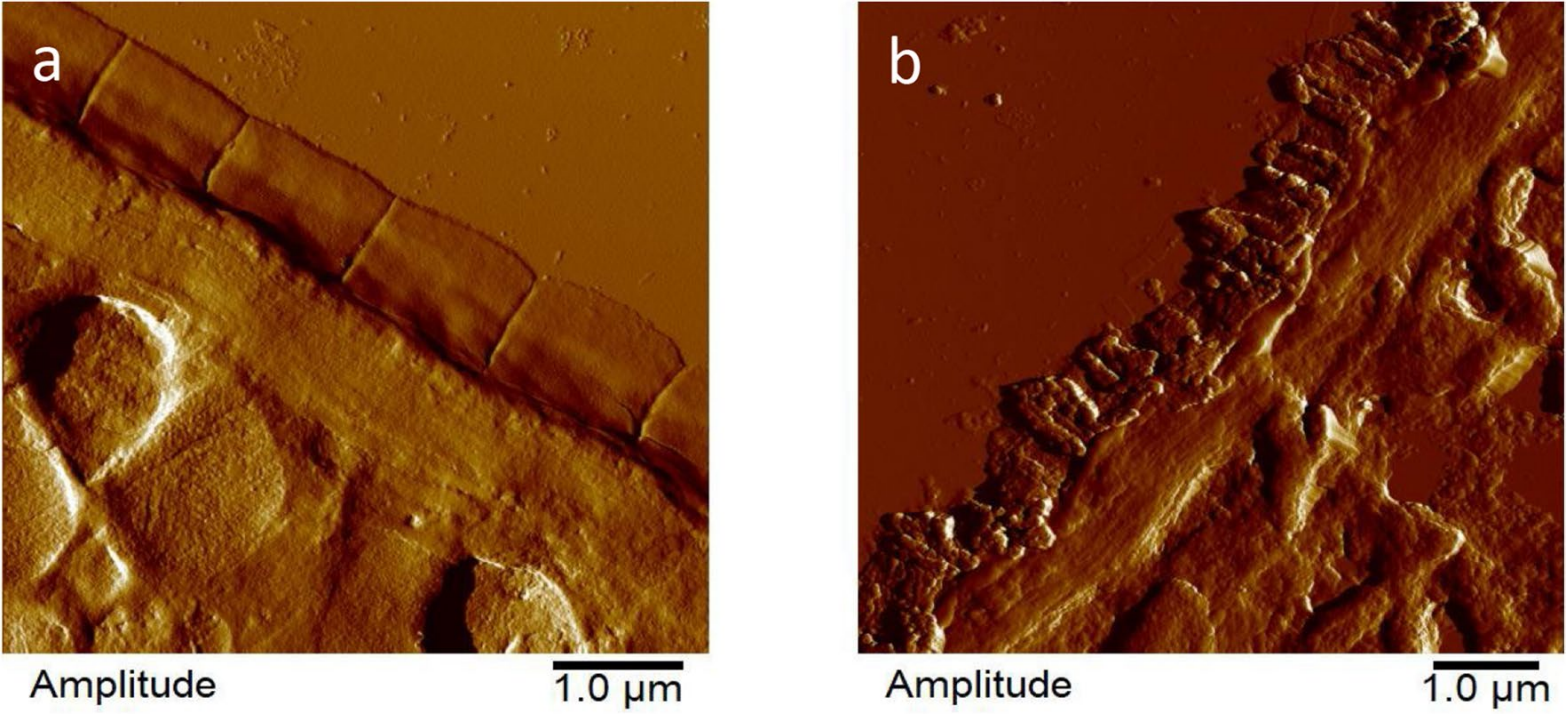
Representative AFM images of 350 nm thick ultramicrotomic sections of young adult *C. elegans* after 24 h of treatment. (**a**) Untreated control (M9 buffer with 1% DMSO). (**b**) Worms treated with CM (2 mg/mL).

Furthermore, to take a more detailed look at the structures underlying the cuticle, AFM imaging of 350 nm thick ultra-sections of embedded *C. elegans* was performed (Fig. 3). Again, the cuticle of the untreated worms showed a regularly spaced pattern of annuli followed by a smooth basal zone, hypodermis and basal lamina attached to the muscle tissue beneath (Fig. 3a). In contrast, the cuticle of the treated samples was completely irregular and agglomerations seemed to have formed throughout the cuticle layer. Attachments to the tissues below were, however, generally still intact, and neither the hypodermis nor the muscles showed a detachment from surrounding tissues or signs of degradation by the treatment (Fig. 3b).

**Figure 4:**
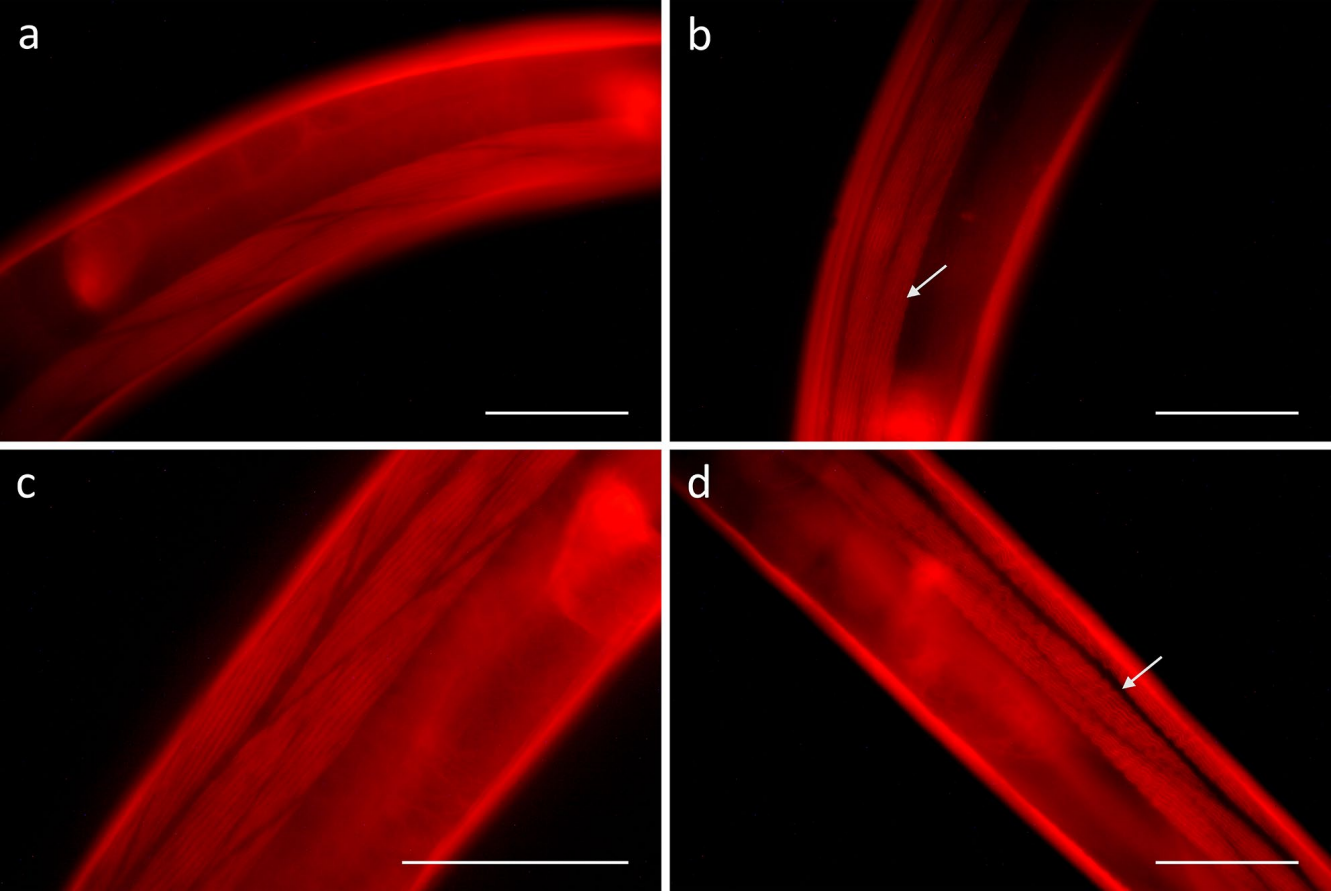
Fluorescence microscopy of young adult *C. elegans* stained with Texas Red-Phalloidin after 24 h and 48 h of treatment in liquid culture, resp., followed by 6 h of regeneration on NGM agar. Left panel: Untreated control (M9 buffer with 1% DMSO) after for 24 h (**a**) or 48 h (**b**). Right panel: Worms treated with CM (2 mg/mL) for 24 h (**c**) or 48 h (**d**). Scalebar 50 µm. White arrows indicate wavy structures of the actin filaments in extract treated worms.

Nevertheless, we stained the actin filaments of the worms’ muscle sarcomeres with Texas Red-Phalloidin, to ascertain that no direct effects on the muscles were responsible for decreased motility. As shown in Fig. 4, wavy structures of the filaments were visible in some of the extract treated worms (Fig. 4c), but not in all individuals. Extending the incubation time to 48 h before 6 h of crawling and staining increased the number of individuals showing such wavy filaments (Fig. 4d) and the effects were more pronounced, however, not all worms were affected and further signs of disruption were not observed.

### Treatment with proanthocyanidins disturbs molting of C. *elegans* larvae

**Figure 5:**
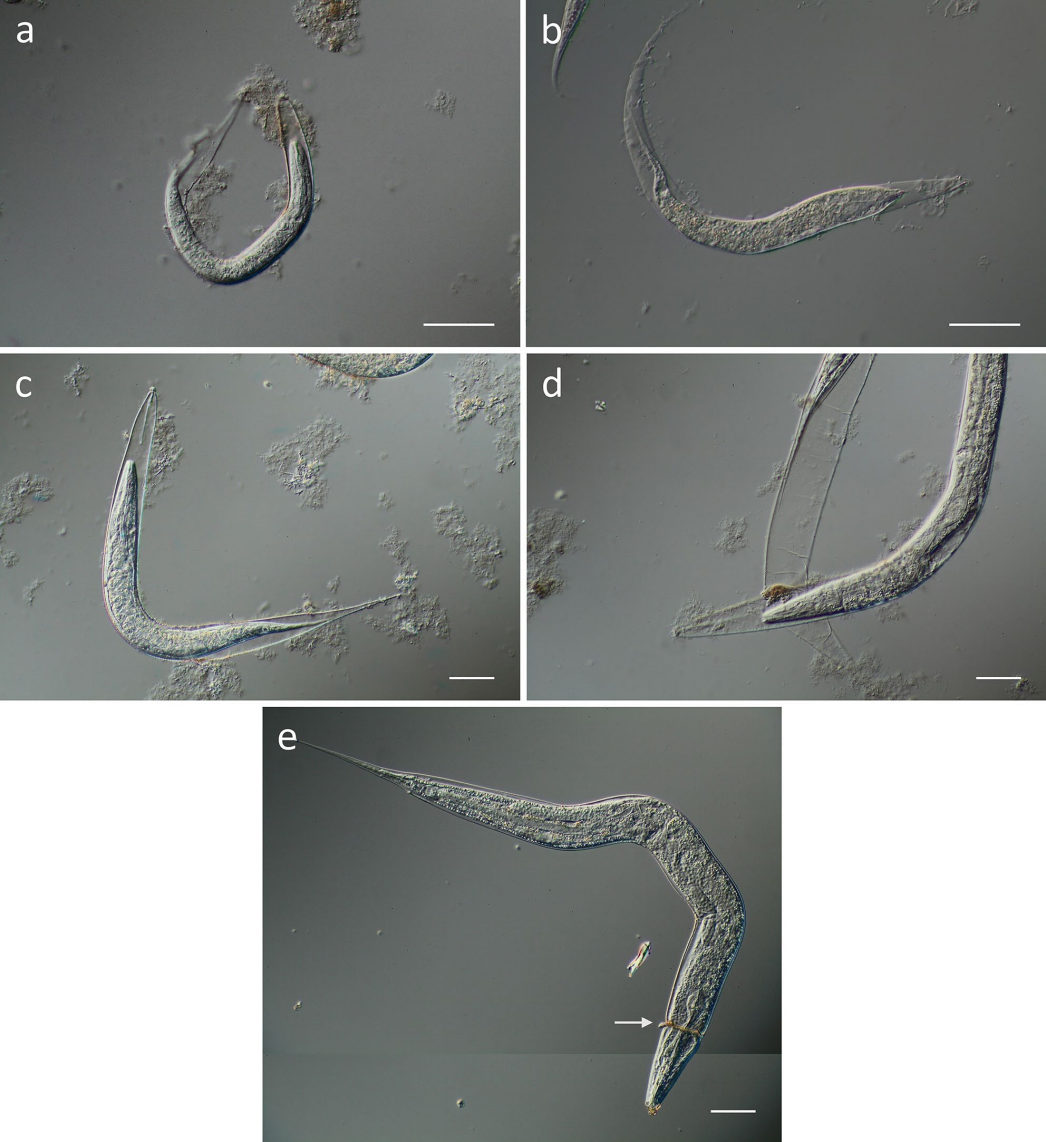
DIC microscopy of different larval stages of *C. elegans* treated with CM (2 mg/mL) overnight. (**a**) L1 larva, (**b**) L2 larva, (**c**) L3 larva, **d**) L4 larva, each entrapped within the unshed cuticle. (**e**) L4 larva with partially shed cuticle causing a constriction in the head region. Scalebar 50 µm.

In addition to young adults, effects of CM in different larval stages were studied, particularly with respect to molting, as the process of exsheathment has frequently been reported to be inhibited by tannins in parasitic nematodes. Larvae at different stages from L1 to L4 were treated overnight with CM (2 mg/mL), so that a molt to the next larval stage would take place during the treatment. As displayed in Fig. 5, the molting process was disturbed by CM treatment in all larval stages. More precisely, a new cuticle was synthesized, but ecdysis was inhibited, entrapping the animals within their old cuticle that was released in the head and tail region. Occasionally, larvae were only partially able to shed their old cuticle which was retained as a constriction around the animals (Fig. 5e).

### The flexibility of the cuticle is impaired by treatment with CM

Considering the disruptions of the cuticle structure with the underlying tissues being only marginally affected, together with the inhibition of larval molt at the stage of ecdysis, we assumed that treatment with CM could impair mechanical properties of the nematodes’ cuticle. Therefore, force spectroscopy experiments were performed on young adult CM treated and untreated *C. elegans* individuals.

**Figure 6:**
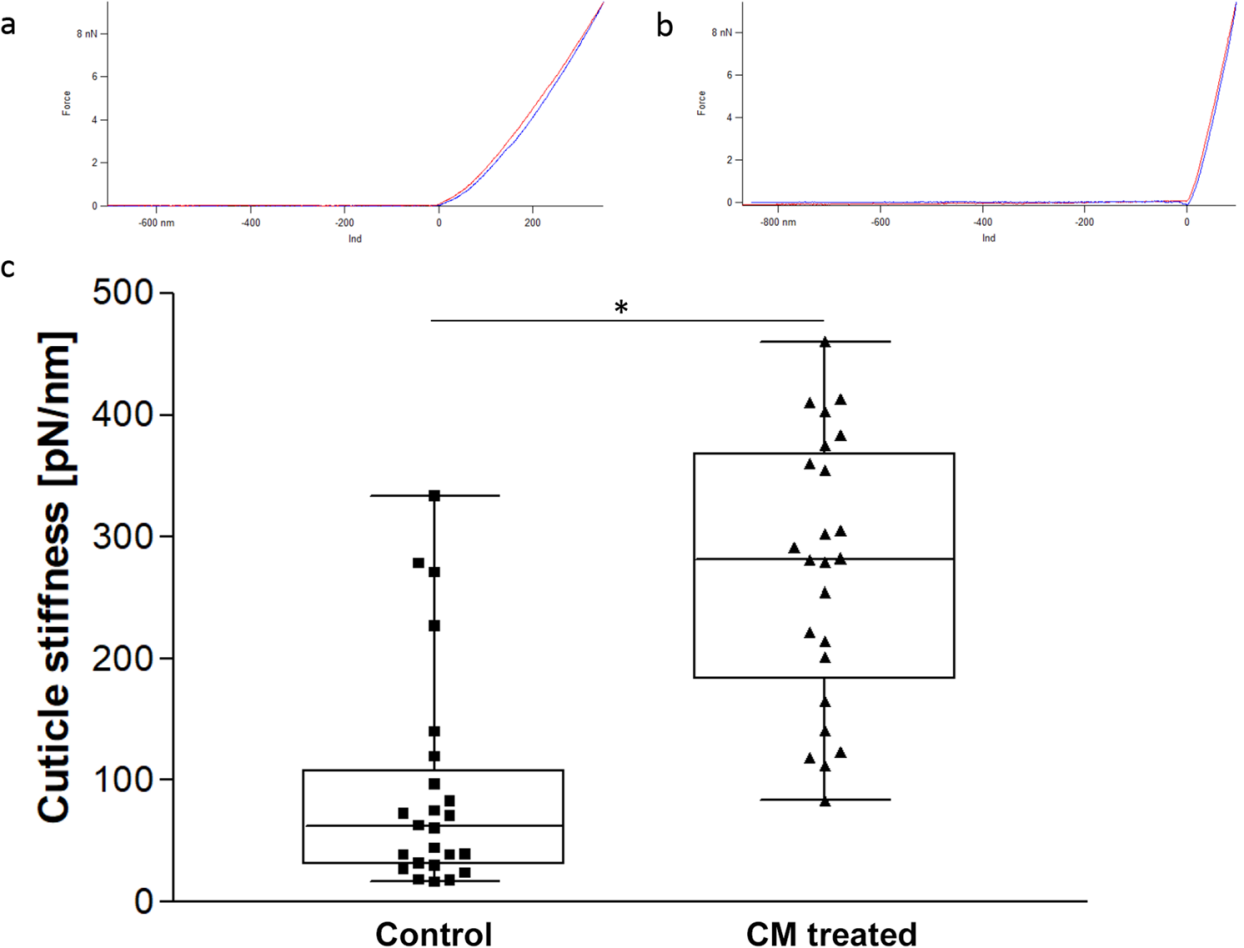
Mean stiffness of single worms measured via AFM force spectroscopy. Every point represents the mean stiffness of a single worm measured at 3 to 10 different positions. Upper panel: Exemplary force-distance curves for a measuring point on an untreated (**a**) and a treated (**b**) worm. The indentation depth is plotted against the applied force. (**c**) Boxplot showing the maximum, the 75% percentile, the median, the 25% percentile and the minimum. The significant upwards shift of the boxplot of treated worms compared to untreated individuals (*p* < 0.001) indicates an increase in cuticle stiffness after CM treatment (2 mg/mL, 24 h).

The cuticle stiffness was evaluated in a total of 24 treated and untreated worms, respectively. Measurements were performed at least on three different parts of the body per worm to include deviations depending on the measured position. Force-indentation curves were generated for each measuring point, in which the indentation depth was plotted against the applied force in order to determine the stiffness in mN/m. Mean values from individual worms were plotted for the untreated versus the CM treated group. As shown in Fig. 6, a clear difference was revealed, indicating that the treatment effected a significant increase in the stiffness of the cuticle.

### Visualization of tannins in C. *elegans*

**Figure 7:**
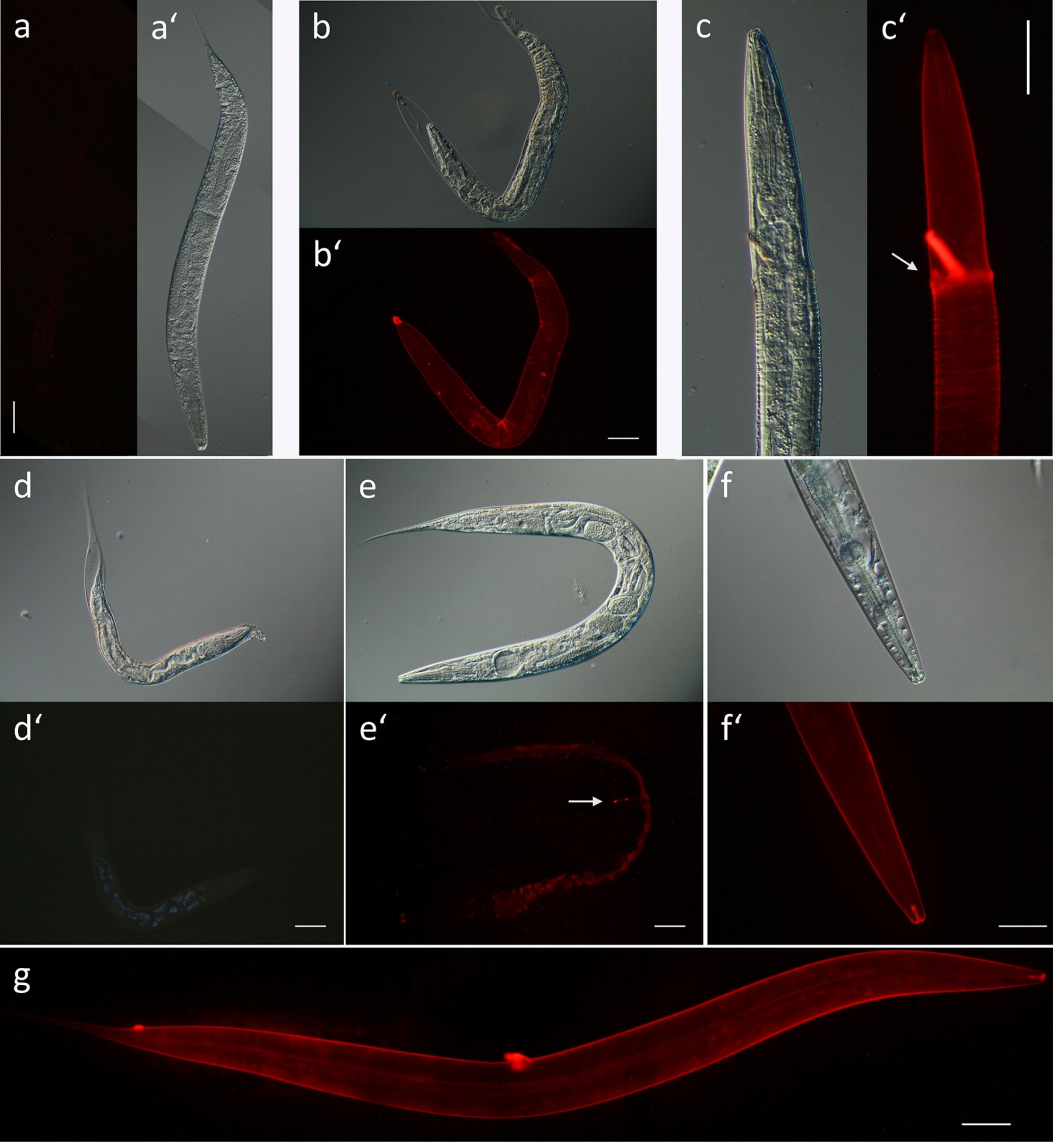
DIC and fluorescence microscopy of *C. elegans* L4 (**a**–**d**) and adult worms (**e**–**g**) after 24 h of treatment. (**a**,**a’**): Untreated control (M9 buffer with 1% DMSO) stained with Wheat Germ Agglutinin, Alexa Fluor 594 Conjugate (WGA). (**b**–**d’**) L4 larvae treated with CM (2 mg/mL) and subsequently stained with WGA (**b’**,**c’**) or fluorescence (λ_ex_ 350/λ_em_ 430 nm) detected without staining (**d’**). Arrow indicates the partially shed cuticle strongly stained with WGA (**c’**). (**e**,**e’**) Untreated adult stained with WGA. Arrow indicates vulva, fluorescence within the worm corresponds to autofluorescence. (**f**,**f’**) Head region of CM-treated worm after WGA staining, showing strong fluorescence in the buccal capsule. (**g**) CM-treated adult worm stained with WGA. Scalebar 50 µm.

To detect a potential accumulation of carbohydrates that may occur due to the molting inhibition ^37^ after treatment of larvae with CM, L4 larvae as well as adult *C. elegans* were stained with WGA-Alexa Fluor 594. In untreated animals, WGA only bound to a small extent in the area of the vulva of adult worms (Fig. 7e,e’), but not at all in larvae (7a,a’). After treatment of L4 larvae with 2 mg/mL CM overnight, molting defects occurred as described earlier and the old cuticle, which could not be removed, was completely stained with WGA (Fig. 7b,b’). Especially in the areas where nematodes were able to break through the old cuticle, WGA seemed to accumulate (Fig. 7c,c’). This could mean that the composition of surface glycans is altered, exposing surface structures that WGA can bind to. On the other hand, it would corroborate the idea that the production of glycans is increased similar to a lubricant^37^, in order to facilitate ecdysis from the rigid cuticle in treated animals. However, unrelated to molting inhibition, WGA also stained the entire cuticle of treated adult worms, especially the buccal cavity, the vulva and the anus (Fig. 7f,f’,g). We therefore assessed whether tannins themselves were also able to bind WGA due to the protein nature of the lectin. Indeed, a fluorescent precipitate was formed from a solution of CM and WGA and thus, we cannot rule out that staining with the WGA conjugate indirectly visualized the procyanidins bound to the nematodes.

To further clarifiy if the staining pattern obtained with the WGA conjugate corresponded to tannins attached to the cuticle, we intended to link the compounds to a fluorescent reagent. Therefore, a purified fraction of CM only containing oligomeric procyanidins was fluorescent labelled using dansyl chloride. Disappointingly, we could hardly detect any fluorescence within the treated worms (2 mg/mL for 24 h) despite the strong yellow fluorescence of the test solution itself. Only the buccal cavity seemed to be weakly stained by the labelled compounds, but not well distinguishable from autofluorescence (Supplementary Fig. S1). However, to our surprise, at the same excitation wavelength we were able to detect orange fluorescent unlabelled procyanidins bound to the nematodes’ cuticle and the buccal cavity, especially at the tip (Supplementary Fig. S1). Moreover, when we examined the intrinsic tannin fluorescence in molting defective larvae, the unshed old cuticle appeared to be brownish fluorescent (Fig. 7d,d’), resembling the staining patterns of the WGA conjugate. This further suggests that WGA staining mainly detected tannins that had bound to the animals during treatment. To confirm that the fluorescence detected in the animals resulted from cuticle-bound tannins that were able to bind to the protein part of the lectin, sites for carbohydrate binding of WGA were blocked by pretreatment with N-acetyl glucosamine, the main ligand for WGA. While the typical fluorescence of the vulva in adult worms observed in the untreated control samples was abrogated by the addition of N-acetyl glucosamine, the staining pattern in the CM treated nematodes remained the same, with or without addition blocking (Supplementary Fig. S2). This suggests that the staining by the WGA conjugate is unrelated to the carbohydrate composition of the cuticle or glycocalyx. As tannins seemed to accumulate at the tip of the animals’ nose as well as in the buccal cavity, we then asked if feeding was affected by CM treatment. Therefore, worms were incubated in CM (1 mg/mL) overnight and subsequently placed on NGM plates containing “pGlo” *E.coli* (HB101 expressing GFP in the presence of arabinose), similar to the procedure described by Raizen et al.^38^. However, no difference in the uptake of bacteria was observed compared to the untreated group (Supplementary Fig. S3).

## Discussion

Aim of the current study was to further investigate effects caused by proanthocyanidins on the cuticle of *C. elegans*, a nematode often used as a model organism to study anthelmintic drug activity. It has been frequently used for genetic investigations, particularly in forward genetic screens, that led to identification of the mode of action of several anthelmintic drugs in the past^33^. While this is a powerful technique to identify target proteins for small molecules, for larger compounds like oligomeric tannins, different miscroscopic methods seemd to be most suitable to detect potential binding sites for this substance class. Due to reasons of compound availability, an ethanol–water (1:1) extract from *C. mucronatum* which does not contain any other anthelmintic constituents apart from oligomeric procyanidins^35^ was used for most of the experiments. In the first step, the cuticle surface was investigated by DIC microscopy. Its pattern was similarly shrivelled compared to the smooth surface of the control group as previously observed via SEM microscopy after treatment of *C. elegans* with fractions of condensed tannins from different plant sources^26^. Longitudinal sections additionally confirmed previous findings showing that in *C. elegans*, tannins disrupt the cuticle structure while the intestine seems obvisouly unaffected^27^. Particularly, high-resolution topographic AFM images of the cuticle surface of living *C. elegans* individuals show that the regular pattern of annuli is hardly recognizable anymore. Such alterations are likely caused by the high affinity of tannins to proline-rich proteins such as the cuticle collagens ^17–19^. Binding and “tanning” the collagenous exoskeleton of the nematodes could affect several functions like cuticle permeability^39^, molting^40^ and locomotion ^18,28^, but also pathways that cuticle components are involved in, e.g. environmental stress response^41^, osmoregulation^42,43^, immune response^44^ or autophagy^45^. More specifically, it is known that components of the extracellular matrix (ECM) can be regulated by transforming growth factor beta (TGF-β)^46^ and in turn, the expression of the *C. elegans* ligand DBL-1 in ventral cord neurons is influenced by the collagen composition of the cuticle^47^. This feedback regulation may be realized by an alteration of biomechanical forces^47^, a crosstalk mechanism which has been frequently reported for human epithelial cells and tissue^48,49^. Therefore, the measured increase in cuticle stiffness could possibly account for differential gene expression in tissues unrelated to the cuticle after tannin treatment^50^.

Regarding the reduced motility observed in tannin-treated adult *C. elegans*^26^, this effect can either be caused by a decreased flexibility of the cuticle^51,52^ or by a direct effect on the worms’ muscles or extracellular matrix components transmitting force to the cuticle^53^. Our microscopic observations in treated worms did not reveal clear visible damages to tissues underlying the cuticle and no detachments of the hypodermis from the cuticle as observed in other parasite species^23,25^, even though animals were transferred from liquid medium to agar plates to increase mechanical forces^36^. Staining of actin filaments only showed mild signs of damage in the form of wavy filaments in some of the treated samples (Fig. 4). Similar to wave-like fibers observed in worms after exercise^54^, such muscle damages could be the result of an increased muscle work necessary for movement within a stiffened cuticle^55^. Experimental evidence to these considerations was provided by AFM force spectroscopy measurements, revealing a more rigid cuticle in the treated nematodes compared to the control group.

Within another study, the effect of tannic acid on the collagenous portion of dentin was investigated and similar to the nematode cuticle, the treatment resulted in increased stiffness^56^. Moreover, not only biomechanical properties of the tissue were enhanced, but also proteolysis of collagens by collagenases was inhibited, either indirectly by an improved stability, or directly by enzyme inhibition^56^. Collagenases belong to the family of matrix metalloproteinases^57^ and in *C. elegans*, two collagen degrading enzymes, NAS-37 and NAS-36, have been reported to be necessary for ecdysis^58–60^. In addition to a decreased motility, inhibition of larval exsheathment has frequently been observed in different parasitic nematodes after exposure to tannins^10^ and in line with these previous reports, treatment with CM inhibited molting in L1 to L4 larvae of *C. elegans*. In general, molting defects can be caused by interference with a variety of proteins^40^. In our case, molting seems to be inhibited at the stage of ecdysis, as larvae are mainly entrapped within their old cuticle which is partially released in the head and tail region. A similar phenotype showing a partial release of the old cuticle at the anterior part of the larva has been found in *qua-1*^40,61^ and *mlt-10* (RNAi)^37^. Both genes are expressed in the hypodermis and fluorescent fusion proteins were found to be secreted to the surrounding tissues^37,61^. In some cases, also the phenotype of *nas-37* mutants was observed, with the old cuticle forming a tight circular constriction around the larvae. Besides an accumulation in the excretory duct and the alae, NAS-37 is located in the cuticle^60^ and could be directly affected by tannin binding, especially since it is predominantly found at the tip of the animals’ nose^60^, a region where tannins seemed to be concentrated (Fig. 7b,b’,f,f’,g). Further, an impact on the hypodermis, not visualized in our experiments, cannot be ruled out, even if the collagenous cuticle seems to be the primary binding site for tannins. Especially the fact that MLT-10 contains a repetitive proline-rich region and that its predicted post-translational modifications are similar to those occuring during biosynthesis of collagen and extracellular matrix proteins^37^, suggests that MLT-10 could also be a target to bind procyanidins. On the other hand, different types of molting defects were observed in CM-treated samples, so the inability to shed the old cuticle may not be related to a specific interference with certain proteins associated with molting. Instead, it seems more likely that it is an effect of a less elastic and hardened cuticle after tannin exposure, i.e. larval movement might not be sufficient to break the old cuticle during ecdysis^40^. As we do not expect large molecules like procyanidins to pass the old cuticle and to affect the new one synthesized underneath, the inability to shed the old cuticle does not seem to arise from a reduced larval motility. Rather, an increased rigidity of the old cuticle that becomes difficult to be mechanically disrupted by the larvae during ecdysis, seems more plausible to explain the observed effects, also because the entrapped larvae were mobile. We finally wanted to explore whether ligands for wheat germ agglutinin became detectable in molting defective CM-treated larvae and worms, similar to the observations in *mlt-10* mutants, to corroborate a potential lubricant function of mucin-like carbohydrates^37^ that could aid in ecdysis. Indeed, shed cuticles of treated L4 larvae were strongly stained by WGA-conjugate, especially at those sites where the cuticle was torn. Moreover, unlike the *mlt-10* mutants, the entire outer (unshed) cuticle of the entrapped larvae was stained, but not the new cuticle underneath (Fig. 7b), probably because of the limited permeability of WGA. However, as mentioned before, an interaction of tannins bound to the cuticle surface with the protein fraction of WGA has to be considered and indeed, a fluorescent precipitate formed after incubation of a CM solution with WGA. Unfortunately, fluorescent labelling of procyanidins by dansylation did not reliably allow the detection of the main binding site of the molecules. However, the unexpected observation that the intrinsic fluorescence of the cuticle-bound procyanidins^62^ within the extract was apparently sufficient for fluorescence microscopy, confirmed the entire cuticle to be covered by the tannins. Additionally, the substances seemed to be concentrated at the vulva, the tip of the nose and in the buccal cavity, particularly in those regions lined by a cuticle (Fig. 7f), i.e. the cheilostom and the buccal capsule^63^ comprising the cylindric buccal prism and at the posterior end the glottoid apparatus with the interradial flaps^63,64^. Fluorescence decreased towards the intestine which could be due to the abscence of collagenous tissue such as the buccal cuticle, but possibly also because of signal quenching within the worm. These findings coincided with similar observations reported in *Haemonchus contortus* after treatment with different tannin-rich plant extracts^20^ and especially with the pattern observed by WGA staining, also after blocking its carbohydrate binding sites with N-acetyl glucosamine, suggesting that the binding sites for tannins were visualized this way. Of note, the *C. elegans* amphid sensilla, involved in chemotaxis, mechanosensation, osmotaxis, and dauer pheromone sensation are located in the labial head region^65^. Most amphid neurons contain ciliated endings of dendrites embedded in a sheath and a socket cell that extend to the outer environment^65,66^. Thus, accumulation of tannins at the nose tip around the lips could impair their neuronal functions. Feeding, however, was not affected by tannin treatment, despite the involvement of the buccal cavity.

In summary, our findings point to the proline-rich collagenous cuticle as the main target structure for anthelmintic proanthocyanidins. The reduced flexibility could be confirmed experimentally and provides a plausible explanation for other anthelmintic effects, such as inhibition of motility and molting as well as mild damages to muscle filaments.

## Methods

### Nematode culture conditions and treatment

*C. elegans* wildtype worms (N2 Bristol strain) were grown and maintained at 20 °C on petri dishes containing standard NGM (Nematode growth medium) agar supplemented with *Escherichia coli* OP50 strain as food source^67^.

Age synchronous worms were obtained using alkaline hypochlorite treatment of adult hermaphrodites for 6 min^68^. Eggs or L1 larvae (hatched overnight in M9 buffer without food source) were seeded on fresh NGM plates supplemented with 800 µL *E. coli* OP50 as a food source and grown at 20 °C until they reached the indicated stage^68^. Before treatment, worms were rinsed off the NGM plates and washed three times with M9 buffer to remove remaining bacteria. Only living worms were included in the experiments. 1% DMSO in M9 buffer was used as untreated control in all assays as it corresponded to the maximum final concentration of DMSO in the test solutions.

### Chemicals and plant extract

If not stated otherwise, chemicals were purchased from Applichem (Darmstadt, Germany). Deionized water (Merck Millipore, Merck, Germany) was used to prepare all solutions. The ethanol–water (1:1) extract from the leaves of *Combretum mucronatum* Schumach. & Thonn. (“CM”) was prepared as described previously^35^. All methods were performed in accordance with the relevant guidelines/regulations/legislation. Information about the collection of plant material is given in^35^, no specific permission was required. The content of tannins was determined according to the method described by the European Pharmacopoeia^69^ in three replicates of 200 mg, resp. For all experiments, a stock solution containing 4 mg/mL *C. mucronatum* extract in M9 buffer was prepared using 2% DMSO as solubilizer. Before use, the solution was centrifuged at 2000 × *g* for 1 min. The final test concentration was obtained by dilution of this stock solution as described for each experiment, respectively.

### DIC microscopy

Differential Interference Contrast (DIC) microscopy was performed with a Leitz/Leica Orthoplan microscope equipped with DIC optics (Leica Microsystems, Wetzlar, Germany). Epifluorescence microscopy of stained worms and larvae was also carried out with a Leitz/Leica Orthoplan microscope equipped with a 200 W HBO-light source and an epifluorescent illuminator using dichroitic filter cubes (A, I2 and N2.1, Leica Microsystems, Wetzlar, Germany).

Young adult worms were treated with 2 mg/mL CM for 24 h, they were then transferred to NGM plates and allowed to move for 6 h before microscopy. For investigation of the molting process, different larval stages (L1, L2, L3 and L4) were treated with 2 mg/mL CM overnight, followed by DIC microscopy without regeneration on NGM-plates.

### Actin staining

Young adult *C. elegans* were treated for 24 h and 48 h with CM at 2 mg/mL and transferred to NGM plates followed by movement for 6 h. Worms were washed three times with M9 buffer and fixed in 4% paraformaldehyde solution for 30 min at room temperature. After fixation, worms were permeabilized with ice cold acetone for two minutes and washed three times with phosphate buffered saline (PBS). The permeabilized worms were incubated for two hours in 3 U/mL Texas Red-X Phalloidin (ThermoScientific, USA) in PBS at room temperature in the dark. Before imaging, the stained worms were washed again three times in PBS^70,71^.

### Processing of microscopic image data

All light microscopic data was processed employing the ImageJ 1.50i^72^ software extension FIJI^73^ (insertion of scale bars, subtraction of background noise in epifluorescence microscopy, adjustment of imaging contrast and brightness in DIC images). Composing of single images was performed using Adobe Photoshop CS6 Ver. 13.0.1 (Adobe Inc., San José, CA, USA).

### Polyethylene glycol embedding and ultra-sectioning

Young adult *C. elegans* were treated for 24 h with CM at 2 mg/mL and transferred to NGM plates allowing movement for 6 h. After treatment, worms were washed with M9 buffer, fixed in 4% paraformaldehyde solution overnight at 4 °C and washed again with M9 buffer. To obtain about 350 nm thick embedment-free ultra-sections of *C. elegans* suitable for atomic force microscopy (AFM) imaging under ambient conditions, samples were embedded in polyethylene glycol (PEG) 4000 as described previously^74^. Briefly, for dehydration of the worms a dilution series of ethanol was performed (15:85, 25:75; 50:50, 70:30, 95:5, 100:0). Worms were incubated twice in each dilution step for 10 min at room temperature under gentle agitation followed by centrifugation for 10 min at 1000 × *g* and removal of the supernatant. The last dehydration step with absolute ethanol was performed three times to ensure that the samples are completely free of water.

The dehydration was followed by PEG 4000 infiltration via an ethanol / PEG dilution series (50:50, 0:100) at 64 °C under gentle agitation. 100% PEG 4000 was applied at least twice to ensure removal of all residual ethanol. Centrifugation with 100% PEG was performed in a pre-heated rotor at 64 °C and 1000 × *g*. After applying the final absolute PEG 4000 step, samples were centrifuged at 64 °C as described above and were subsequently allowed to solidify under ambient conditions in conical 1.5 mL Eppendorf reagent tubes. Employing the mentioned embedding technique, solidified specimen blocks resulted, containing around 500–1000 worms in their apexes. The specimen blocks were afterwards removed in one piece from the reagent tubes and melted onto an aluminium base to ensure sufficient mounting capabilities in the ultra-microtome employed for sectioning (Leica/Reichert Ultracut E, Leica Microsystems, Wetzlar, Germany).

Ultra-sectioning into slices of approx. 350 nm followed by dehydration and immobilization was performed as described previously^74^. Ultra-sectioning of the sample blocks was carried out on a Leica/Reichert Ultracut E microtome (Leica Microsystems GmbH, Wetzlar, Germany) with typical glass knifes, freshly prepared on a LKB Bromma 7801B KnifeMaker (LKB Bromma, Stockholm, Sweden). Bands of 350 nm thick ultra-sections were yielded under ambient conditions employing an inclination angle of 3 degrees and section velocities around 50 mm/s. Bands containing around 200 single sections were afterwards transferred into a 50 µL drop of H_2_O on a poly-L-lysin-coated glass slide (Polysine slides, Gerhard Menzel GmbH, Braunschweig, Germany) and a coverslip was gently applied. The samples were subsequently allowed to rest for at least five minutes in order to mediate the adherence of the ultra-sections to the coated glass slides. The coverslip was afterwards gently washed away and the immobilized ultra-sections were quickly dried under pressurized airflow (hand bellows) before imaging under ambient conditions by intermittent contact mode AFM.

### Atomic force microscopy of C. *elegans* ultra-sections under ambient conditions

Imaging of immobilized and dehydrated *C. elegans* ultra-sections was carried out employing intermittent contact mode under ambient conditions using a Bruker Bioscope I AFM equipped with a Nanoscope IIIa controller and soft n-type silicon cantilevers (HQ:NSC14 Al BS, µmesh, Sofia, Bulgaria). Imaging parameters were as follows: oscillation 10% below determined resonance frequency, free RMS amplitude of around 1 V with setpoint values around 0.7 V and a scanning rate of 0.5 Hz.

### Processing of AFM image data

AFM image data was processed employing the software Nanoscope Analysis 1.5 (Bruker, Karlsruhe, Germany) in case of Fig. 3 and Igor Pro 6.3.8.1 (Asylum Research, Santa Barbara, California, USA) in case of Fig. 2. Typically, flattening (0th and 1st order algorithms) and crop operations were carried out to improve depiction of image data. In case of tilt artefacts, plane fit operations were applied to create a planar image profile.

### Sample preparation for cuticle imaging and force spectroscopy measurements under liquid conditions

For AFM experiments under liquid conditions, samples were prepared according to Essman et al.^75^ Briefly, after 24 h of incubation, treated (2 mg/mL CM) as well as untreated, young adult worms were paralyzed in a solution of 10 mg/mL of 2,3-butanedione monoxime for 1 h and subsequently transferred to an 4% agarose pad on a cover slide, which was previously epoxy-glued onto a small magnetic steel disc. Worms were afterwards immobilized on head and tail with a cyanoacrylate-based tissue glue (Vetbond, 3 M Animal Care Products, USA) to allow reliable AFM operation and immediately covered with M9 buffer to prevent dehydration.

### Topographical cuticle imaging under liquid conditions

AFM-based topographical imaging of the cuticle morphology of living, paralyzed and immobilized *C. elegans* individuals was carried out employing an Asylum Research Cypher-S AFM (Asylum Research, Santa Barbara, USA), equipped with pyrex nitride probes of triangular shape (PNP-TR cantilever 1, NanoWorld, Neuchâtel, Switzerland) in intermittent contact mode under M9 buffer (RMS amplitudes around 0.8 V and setpoint amplitudes around 0.6 V).

### AFM-based force spectroscopy of the cuticle of living C. *elegans* individuals

Force spectroscopy measurements were carried out on living immobilized worms employing an Asylum Research Cypher-S AFM equipped with pyrex nitride probes of triangular shape (PNP-TR, cantilever 1, NanoWorld, Neuchâtel, Switzerland). The individual spring constant of each cantilever was determined prior to measurements employing the thermal noise method implemented in the Cypher-S measurement software (Spring Constant Tutor algorithm; starting with the determination of the inverse optical laser sensitivity (InvOLS) by recording multiple contact mode force plots in air and averaging resulting InvOLS values; subsequently a series of thermal tunes were recorded with a withdrawn tip in air, allowing fitting of the averaged thermal tunes to the individual resonance frequency of the used cantilever according to the equi-partition theorem, subsequently allowing the calculation of the individual spring constant of each employed cantilever; finally, after adding measurement buffer, invOLS were updated in liquid by recording a series of force distance plots on a hard glass surface and averaging the resulting slopes).

Around 50 force distance curves with calibrated probes on paralyzed and immobilized *C. elegans* were recorded at 3–10 positions on every individual worm (scan rate 1 Hz, force distance 1 µm, deflection trigger point 50 nm and velocity of 2 µm/s), avoiding eggs, since in far-developed eggs movement of larvae could potentially interfere with the measurement. The loading curves of the force distance plots were afterwards fitted according to the Oliver-Pharr model allowing the calculation of the stiffness in mN/m (Igor Pro 6.3.8.1, Asylum Research, USA). Resulting stiffness values were averaged for each worm allowing a direct comparison between treated and untreated worm individuals. Statistical analysis using an unpaired two-tailed t-test was carried out using GraphPad Prism Ver. 3 (GraphPad Software, Inc., La Jolla, CA, USA). Occasionally, measured values considerably exceeded the average range in both groups and we assume that in this case, the cantilever had contact with the tissue glue that was used to immobilize the worms. Thus, values that deviated from the mean by more than three standard deviations (z-score > 3) were considered outliers and excluded. This phenomenon occurred in one animal per treatment group, so that finally, a total of 24 untreated and 24 treated worms were included in the evaluation, respectively.

### Wheat germ agglutinin staining

A stock solution of 1 mg/mL, Wheat Germ Agglutinin (WGA) Alexa Fluor 594 Conjugate (Invitrogen, USA) in water and a stock solution of 125 mg/mL N-acetyl glucosamine (Aldrich Chemical Company Inc., Milwaukee, WI, USA) in M9 buffer were prepared. Age synchronous L4 larvae and young adult *C. elegans* were treated overnight with 2 mg/mL CM, respectively. After treatment, worms were washed three times with M9 buffer. Washed worms were transferred into 100 µL of a 50 µg/mL dilution of WGA Alexa Fluor 594 conjugate in M9 buffer in a 96-well plate and incubated for 1 h at room temperature in the dark under gentle agitation^37^. Before imaging, the stained worms were washed two to three times in M9 buffer. 100 µl of a solution of CM in M9 buffer were mixed with 50 µg/mL WGA Alexa Fluor 594 conjugate and incubated for approx. 1 h at room temperature with shaking. The suspension was then centrifuged at 20,000 × *g*. The pellet was washed twice with M9 buffer and centrifuged again. The pellet was then resuspended in approx. 15 µL M9 buffer and examined by epifluorescence microscopy. In parallel, a control without CM was prepared in the same way to check whether the WGA Alexa Fluor 594 conjugate also forms precipitates without tannin addition. For blocking the carbohydrate binding sites of WGA, N-acetyl glucosamine was added to a dilution of WGA Alexa Fluor 594 conjugate in M9 buffer to a final concentration of 50 µg/mL WGA Alexa Fluor 594 conjugate and 25 mg/mL N-acetyl glucosamine in 100 µl. The mixture was preincubated for 1 h before adding the worms^76,77^. The further staining procedure was then performed as described above.

### Dansylation of procyanidins and staining

12.5 mg of a fraction purified from CM that contained only procyanidins^35^ were dissolved in 50 mL acetone-H2O 8:2 under nitrogen. 2.5 mg dansyl chloride were added and the pH of the solution was raised to approx. 10 by addition of an 8% solution of NaHCO3 in water. The solution was heated to 75 °C under reflux cooling for 10 min while stirring. Acetone was removed under vacuum and the aqueous suspension was lyophilized. The outcome was monitored by thin layer chromatography (stationary phase: silica gel 60 F254; mobile phase: diethyl ether/acetone (20: 2.5 v/v); detection at 366 nm), since only the product is fluorescent. Protein binding properties were verified by suspending 2 mg of the product obtained in 5 mL H2O together with 10 mg hide powder (Sigma–Aldrich, Steinheim, Germany). The suspension was shaken for 1 h at 70 rpm in the dark and was then centrifuged at 6000 rpm for 5 min. The supernatant as well as a fresh solution of the dansylated fraction were run on a TLC as described above and fluorescence was significantly reduced. Young adult worms were treated with 2 mg/mL of dansylated procyanidins from CM in M9 buffer for 24 h. Before microscopy, worms were washed up to three times with fresh M9 buffer to remove residual solution of dansylated OPC.

### Feeding inhibition

Bacteria (*E.coli* HB101) were transformed according to the manufacturer’s manual using the pGLO Bacterial Transformation Kit (BioRad, München, Germany). Cultures were kept on LB agar containing ampicillin (50 μg/mL). Prior to the assay, liquid overnight cultures were started which were supplemented with arabinose (6 mg/mL medium) to induce GFP expression. NGM plates (2.5 cm) were seeded with 80 μL of the liquid overnight culture and allowed to grow for 3 h at 37 °C. Worms of the untreated control (DMSO 1%) and the treated group (CM at 1 mg/mL for 18 h) were transferred to an NGM plate seeded with “pGLO”, respectively and were allowed to feed for 3 h. For microscopy, nematodes were anaesthetized in a drop of levamisole-HCl solution (100 mmol/L), covered by a cover slip and bacterial fluorescence was assessed using a confocal microscope (Zeiss LSM 510, Carl Zeiss Microscopy, Jena, Germany).

## Supplementary Information

Supplementary Information 1.

Supplementary Information 2.

## Data Availability

All raw data for determination of the cuticle stiffness, including the force-distance curves, and for determination of the tannin content are provided as supplementary material.
