# Supplementary figures and images for: Condensed tannins act as anthelmintics by increasing the rigidity of the nematode cuticle
Source: Sci Rep. 2022 Nov 7;12:18850. doi: 10.1038/s41598-022-23566-2 (PMC9640668; doi:10.1038/s41598-022-23566-2)

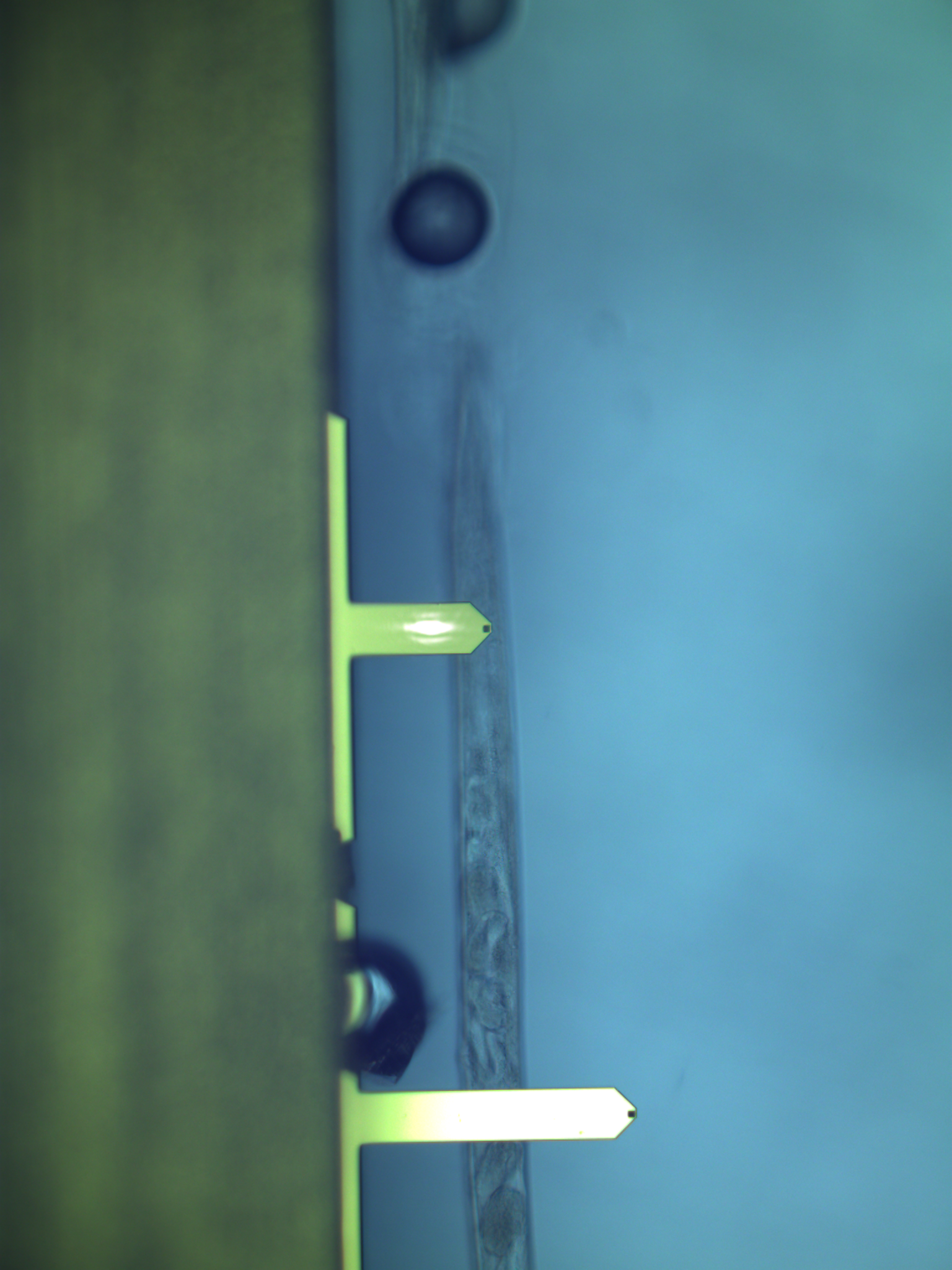

Supplement: Supplementary file 1 — Supplementary Information 1. [file 41598_2022_23566_MOESM1_ESM.zip › Force Spectroscopy Data/Force-indetation curves/treated CM/replicate 1/1.1/Image0388.tif]

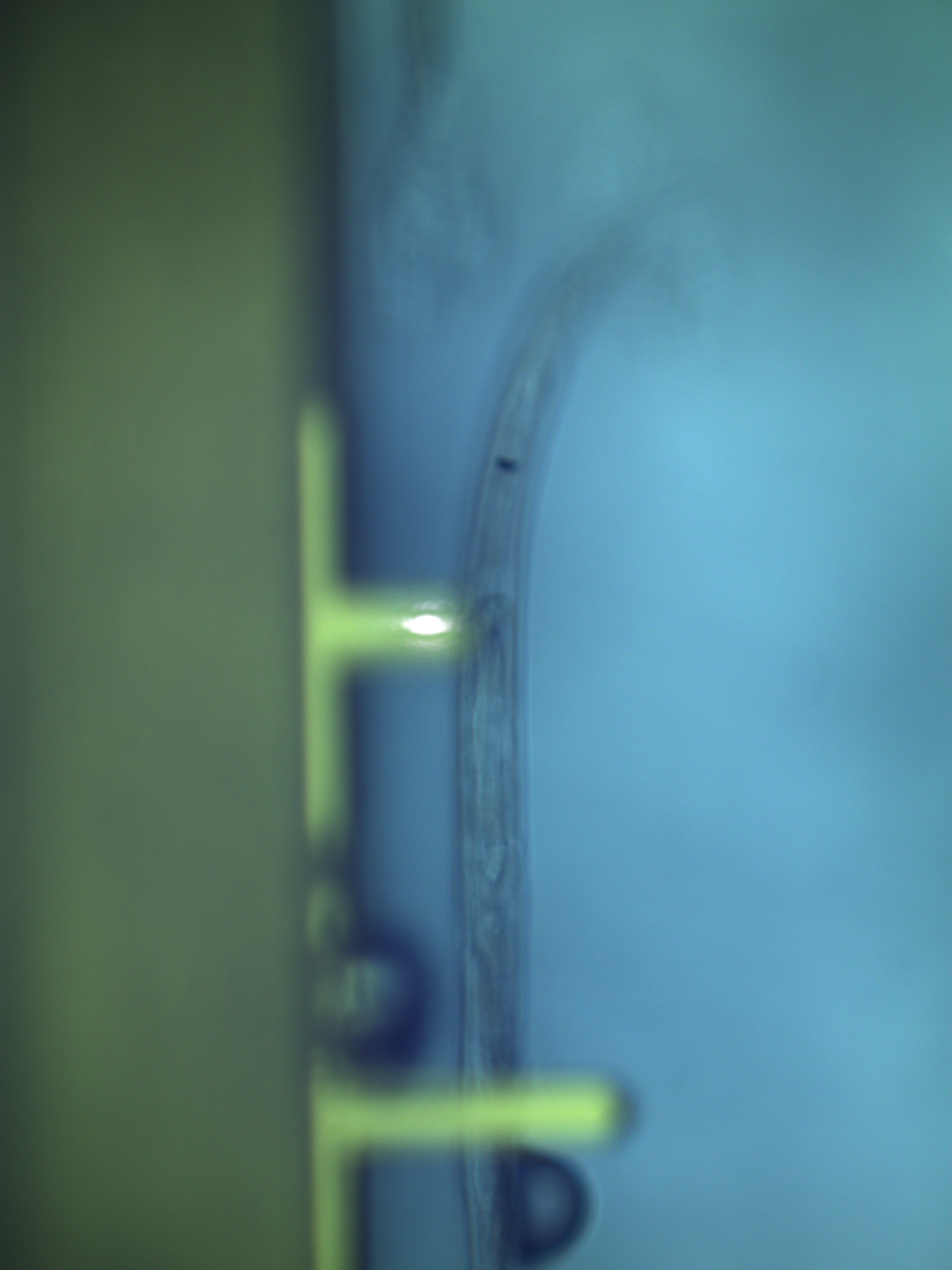

Supplement: Supplementary file 1 — Supplementary Information 1. [file 41598_2022_23566_MOESM1_ESM.zip › Force Spectroscopy Data/Force-indetation curves/treated CM/replicate 1/2.1/Image0389.tif]
