# Supplementary material for: Condensed tannins act as anthelmintics by increasing the rigidity of the nematode cuticle
Source: Sci Rep. 2022 Nov 7;12:18850. doi: 10.1038/s41598-022-23566-2 (PMC9640668; doi:10.1038/s41598-022-23566-2)
Supplement: Supplementary file 2 — Supplementary Information 2. [file 41598_2022_23566_MOESM2_ESM.pdf]

Supplementary Data

Condensed tannins act as anthelmintics by increasing the rigidity of the nematode cuticle

Luise Greiffer[1], Eva Liebau[2], Fabian C. Herrmann[1†], Verena Spiegler[1†*]

[1]Institute for Pharmaceutical Biology and Phytochemistry, University of Münster, Münster, Germany

[2]Institute of Integrative Cell Biology and Physiology, University of Münster, Münster, Germany

[†]These authors have contributed equally and jointly supervised the work

*Corresponding author

E-mail address: verena.spiegler@uni-muenster.de

Fluorescent microscopic visualization of procyanidins bound to *C. elegans*

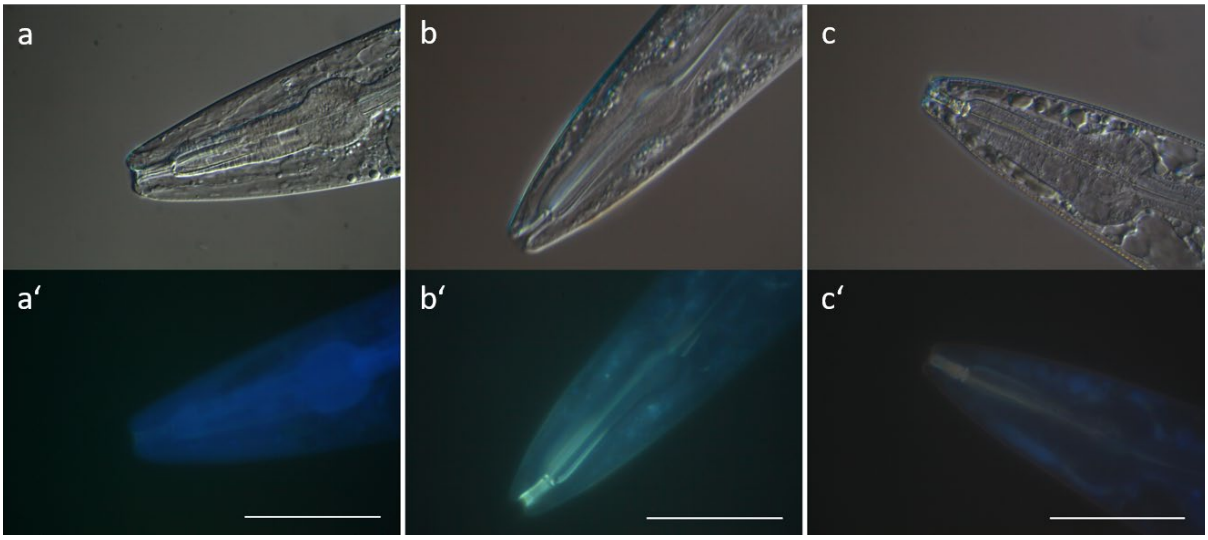

Fig. S1: DIC (upper panel) and fluorescence microscopic images (λ_{ex} 350 / λ_{em} 430 nm; lower panel) of the head region of *C. elegans* adult worms after 24 h of treatment. (**a**, **a'**): Untreated control. (M9 medium with 1% DMSO). (**b**, **b'**) Samples treated with dansylated procyanidins from CM (2 mg/mL). Weak yellow fluorescence was detected in the buccal cavity and cuticle at the anterior end of the worm. (**c**, **c'**) Treated with CM (2 mg/mL). Orange-brown fluorescence was observed in the buccal cavity and the cuticle. Scalebar 50 µm.

Blocking binding sites of wheat germ agglutinin with N-acetyl glucosamine does not affect the staining pattern in L4 larvae and adult worms

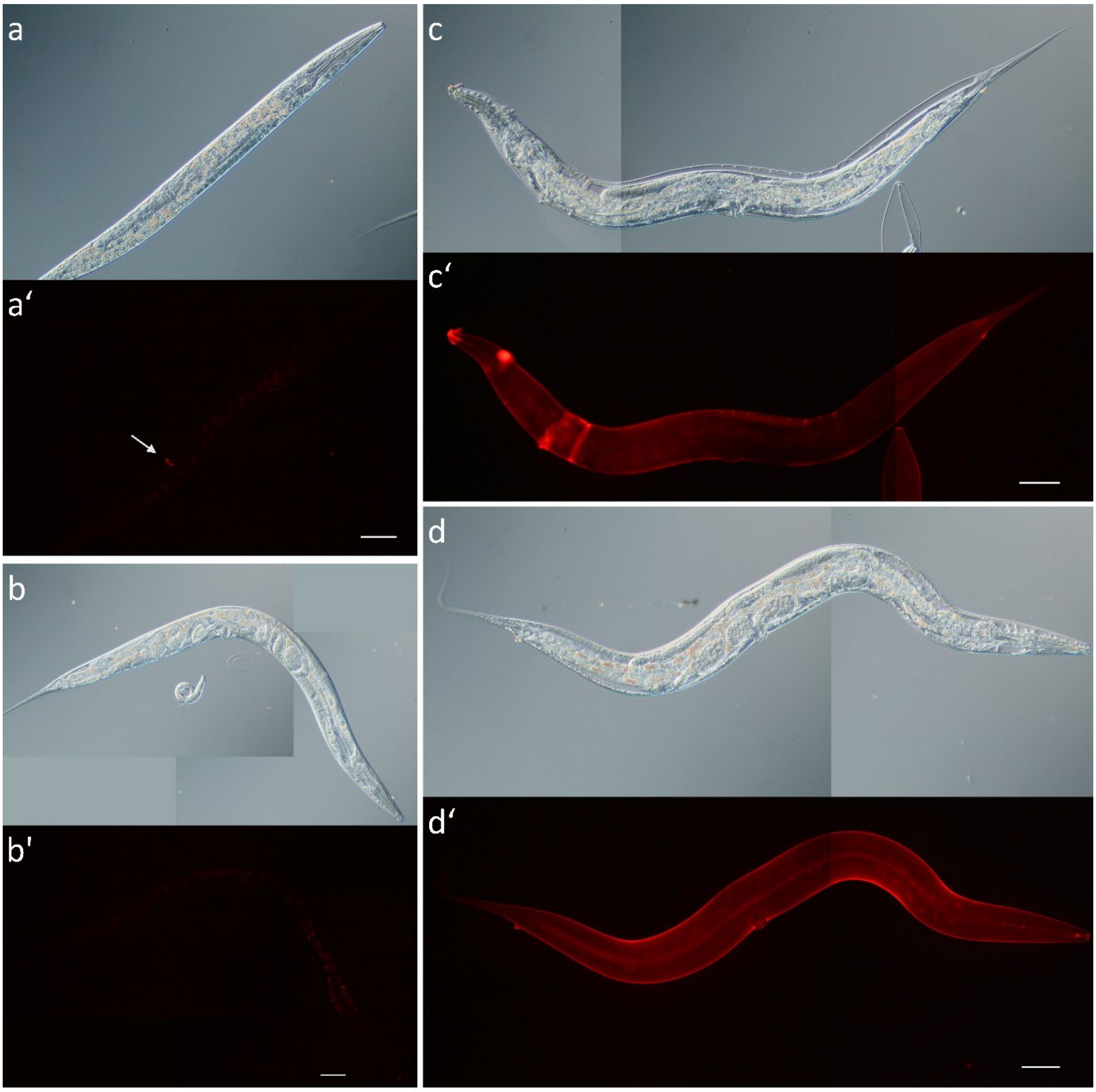

Fig. S2: DIC and fluorescence microscopy of *C. elegans* adult worms (**a,b,d**) and L4 larvae (**c**) after 24 h of treatment. (**a**,**a'**) Untreated control (M9 buffer with 1% DMSO) stained with Wheat Germ Agglutinin, Alexa Fluor 594 Conjugate (WGA). Arrow indicates fluorescent vulva of the worm. (**b,b'**) Untreated control stained with WGA after blocking its carbohydrate binding sites with N-acetyl glucosamine (GlcNAc). (**c'**,**d'**) Animals treated with CM (2 mg/mL) and subsequently stained with GlcNAc-blocked WGA. Scalebar 50 µm.

Treatment with *C. mucronatum* extract does not inhibit feeding

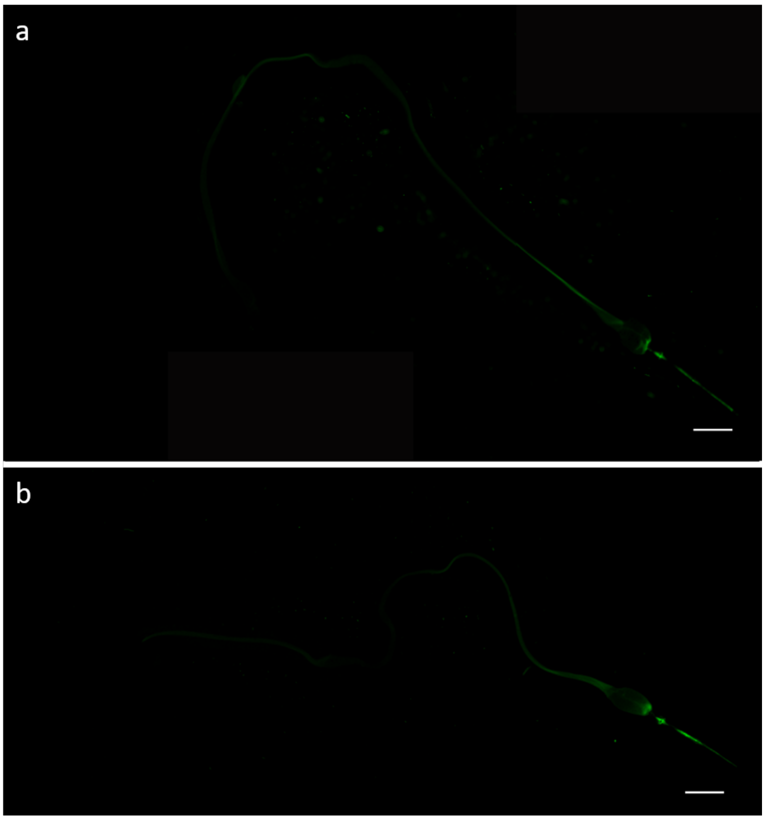

Fig. S3: Representative images showing adult *C. elegans* after overnight treatment and feeding "pGlo" *E.coli* HB101 for 3 h. (**a**) Untreated control, (**b**) worm treated with CM (1 mg/mL). Bacteria expressing green fluorescent protein can be observed similarly in both groups in the anterior half of the nematodes and to a lesser extent throughout the intestine. Scalebar 50 µm.
